# Polymorphisms within Fas gene are not associated with occult hepatitis B virus infection: Polymorphisms within Fas gene in occult HBV infection

**Published:** 2011-01-01

**Authors:** Mohammad Kazemi Arababadi, Adel Mohammadzadeh, Ali Akbar Pourfathollah, Derek Kennedy

**Affiliations:** 1 Department of Microbiology, Hematology and Immunology, Faculty of Medicine, Rafsanjan University of Medical Sciences, Rafsanjan, IR Iran; 2 Molecular-Medicine Research Center, Rafsanjan University of Medical Sciences, Rafsanjan, IR Iran; 3 Department of Immunology, School of Medical Sciences, Tarbiat Modarres University, Tehran, IR Iran; 4 School of Biomolecular and physical Science, Eskitis Institute for Cell and Molecular Therapies, Griffith University Nathan, Queensland, Australia

**Keywords:** Hepatitis B infection, Fas, Polymorphism, HBsAg, HBV, DNA

## Abstract

**Background:**

Occult hepatitis B infection (OBI) is a form of hepatitis in which there is an absence of detectable HBsAg, despite the presence of HBV-DNA in the peripheral blood of patients. It seems that non-effective or attenuated immune system responses against HBV lead to the development of OBI. Previous studies showed that the Fas/Fas ligand (FasL) system is an important death signaling pathway that is used by cytotoxic T lymphocytes to eradicate HBV from the liver.

**Objectives:**

To investigate polymorphisms in the -670 region of the Fas gene in those with OBI.

**Patients and Methods:**

The plasma samples from 3700 blood donors were tested for HBsAg and anti-HBs by ELISA. The HBsAg-/anti-HBc(+) samples were selected and screened for HBV-DNA by PCR. Those with HBV-DNA were diagnosed as OBI and PCR-RFLP technique was performed to examine polymorphisms within their Fas gene.

**Results:**

352 (9.5%) of 3700 blood samples were HBsAg-/anti-HBc(+). HBV-DNA was detected in 57 (16.1%) of 352 HBsAg-/anti-HBc(+) samples. Therefore, 57 HBsAg-/anti-HBc+/HBV-DNA(+) patients were diagnosed as OBI. Patient and control groups had no significant differences in terms of the studied polymorphisms.

**Conclusions:**

The functional polymorphisms in the promoter region of Fas gene are not associated with OBI. Therefore, it may be concluded that polymorphisms at the -670 position of the Fas gene do not have any critical effects on the immune response against HBV in OBI.

## Background

Occult hepatitis B infection (OBI) is a clinical form of hepatitis B in which there are no detectable HBsAg in patient's serum, despite being positive for HBV-DNA [1]. Some of the patients do not have detectable HBV-DNA in serum, despite the presence of HBV-DNA in hepatocytes [2]. Furthermore, high prevalence rates of OBI in chronic HCV-infected patients [3], hemodialysis patients [1][4], immunocompromised patients [5] and HIV-infected patients [6] have been reported. OBI is clinically important because it may induce chronic liver disease [2] and cryptogenic cirrhosis [7]. This type of hepatitis also poses a threat to blood transfusion services and its detection remains a significant challenge for these agencies. The high prevalence of OBI in Iranian blood donors [8][9][10] may be a critical risk for post-transfusion hepatitis (PTH), and despite appropriate screening of all donated blood and blood components for HBsAg, some cases of PTH B are reported [8][11]. The majority of PTH B infections are caused by OBI [12] which we previously reported in our investigations in Isfahan [9] and Kerman [10], the two central provinces of Iran. The mechanisms responsible for progression of OBI are yet to be clarified however, some investigators have suggested that genetic and immunological parameters may play a significant role in the resistance of some individuals and sensitivity of other patients [11][13][14]. Previous studies showed that the Fas/Fas ligand (FasL) system is an important death signaling pathway that is used by cytotoxic T lymphocytes to eradicate HBV from the liver [15]. Elevated expression of FasL was also reported during HBV infection by some investigators [16][17]. Furthermore, several studies showed that the polymorphisms within the Fas Fas (-670 A→G) gene can alter its expression [18][19].

## Objectives

This study was conducted to investigate the relation between OBI and the functional polymorphisms within the promoter region of Fas gene.

## Patients and Methods

### Patients

Peripheral blood samples were collected from 3700 volunteer blood donors of the Rafsanjan Blood Transfusion Services (Kerman, Iran) and placed in EDTA pre-coated 5.5-mL tubes. The samples were centrifuged at 3700 g for 4 min and the sera were collected. All sera were separated within 24 hrs of collection. If needed, serum samples were stored at 20 ºC for a maximum of two months or at 70 ºC, when longer storage was required for further processing. For analysis of polymorphisms a 2-mL aliquot was collected from patients with OBI (57 cases) and 100 healthy controls (HBsAg-/anti-HBc+/HBV-DNA+). The study protocol was approved by the Ethical Committee of Rafsanjan University of Medical Sciences. All of the participants completed and signed an informed consent form which was designed based on the objectives of the study.

### Detection of serological HBV markers

HBsAg screening tests were performed by enzyme-linked immuno-sorbent assay (ELISA) (Behring, Germany). Anti-HBc screening tests were performed by a manual microplate enzyme immunoassay using an anti-HBc commercial kit (RADIM, Italy). The present method is based on a competitive enzyme immunoassay (EIA). All of the samples were also screened by ELISA (RADIM, Italy) for possible HCV, HIV and HTLV-1 infections.

### HBV-DNA extraction from plasma samples

Viral DNA was purified from 200-µL of plasma samples. Briefly, each plasma sample was incubated at 72 ºC for 10 min and then cooled down to 4 ºC for 5 min in 200 µL proteinase K (200 µg/mL) (Cinnagen, Iran). Following phenol/chloroform extraction (1:1), the viral DNA was precipitated with ethanol and the pellet was re-dissolved in DNase-free, deionized water (Cinnagen, Iran) and stored at 20 ºC for further use.

**Table 1:** The sequence of the primers used in the study as well as the appropriate annealing temperatures and expected PCR product sizes

**Target Genes**	**Primers**	**Annealing temperature**	**Product size **(bp)
**S gene **(HBV)	F: 5' TCGTGGTGGACTTCTCTC 3'	60 °C	500
	R: 5' ACAGTGGGGGAAAGCCC 3'		
**Fas **(‑670)	Fas-670 F:5' CTACCTAAGAGCTATCTACCGTTC 3'	58 °C	233
	Fas ‑670 R: 5' GGCTGTCCATGTTGTG GCTGC 3'		

### HBV-DNA polymerase chain reaction (PCR) and gel eletrophoresis

PCR was carried out in a 25-µL mixture containing 10 mM tris-HCl (pH 8.3), 50 mM KCl, 1.5 mM MgCl2, 0.01% gelatin, 5 units recombinant TaqDNA polymerase, 200 µM of each dNTP, 0.6 µM of each primer, and 5 µL of the DNA extracted from 200 µL of plasma. The sequences of all primers used in this study are shown in Table 1. For HBV analysis, the primers are designed to amplify a 500-bp amplicon of the surface antigen or S gene of the HBV genome. Fast temperature cycling was performed using a thermal cycler (C1000, Bio-Rad, USA). PCR amplification was performed using the touchdown method which included one cycle of 93 ºC for 60 sec, 60 ºC for 20 sec and 72 ºC for 40 sec, then five cycles of 93 ºC for 20 sec, 60 ºC to 56 ºC for 20 sec and 72 ºC for 40 sec followed by 30 cycles of 93 ºC for 20 sec, 55 ºC for 20 sec and 72 ºC for 40 sec. HBV genomic DNA provided by the Cinnagen company (Iran) and a negative patient sample were used as positive and negative controls, respectively. For the analysis of the PCR amplification, 10 µL of the amplified DNA were run on 2% agarose gel after addition of 4 µL of loading buffer using a chemi-doc-XRS system (Bio-Rad, USA). The presence of a 500-bp fragment indicated a positive result. In parallel with samples, a 100-bp DNA ladder was also run on the gels to estimate the molecular weight of DNA fragments in the gel.

### Genomic DNA extraction

Peripheral blood was collected in EDTA tubes and genomic DNA was extracted using a commercial kit (Bioneer, Korea) following the recommended procedures. Extracted DNA was aliquoted (for each patient sample) and stored at 20 ºC for further use.

### Detection of polymorphisms

The gene polymorphisms were analyzed by the PCR-restricted fragment length polymorphism (PCR-RFLP) method. PCR of the Fas gene was performed in a volume of 50 µL containing 250 ng of DNA template, 200 µM of each dNTP (Cinnagen, Iran), 0.5 U Taq DNA polymerase (Cinnagen, Iran), 1x PCR buffer (Cinnagen, Iran), 3 mM MgCl2, and 5 pM of each specific primer (Table 1). The PCR conditions were; an initial denaturation at 95 ºC for 5 min, followed by 35 cycles of melting at 95 ºC for 50 sec, suitable annealing temperature for 50 sec (Table 1), and extension at 72 ºC for 50 sec, with a final extension step of 5 min at 72 ºC using a thermal cycler (C1000, Bio-Rad, USA). The expected size of the amplified PCR product used to detect the -670 amplicon of Fas was 233 bp. The ScrFI restriction enzyme (Fermantase, Finland) was used to distinguish the Fas -670 A→G polymorphisms, which resulted in 189 plus 44-bp fragments in the case of the -670 G allele. More than 10% of the samples were randomly selected and retested by appropriate PCR-RFLP techniques for confirmation; the results were 100% concordant. The digested products were run on a 2.5% agarose gel (Cinnagen, Iran) and analyzed using a chemi-doc-XRS system (Bio-Rad, USA) after staining with ethidium bromide.

### Statistical analysis

Hardy-Weinberg equilibrium was assessed using genotype data. Allele and genotype frequencies were calculated in patients and healthy controls by direct gene counting. Statistical analysis of the differences between groups was determined by x(2) test using EPI 2000 and SPSS® ver 13. A p < 0.05 was considered statistically significant.

## Results

**Figure 1:**
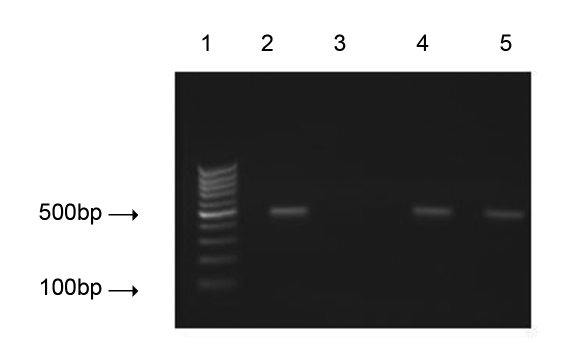
HBV-DNA PCR. An ethidum bromide stained agarose gel showing typical results of HBV-DNA PCR screening of collected samples which were HBsAg negative and anti-HBc positive. Lane 1: DNA ladder marker; lane 2: A positive control showing the expected 500-bp product; lane 3: A negative control; lanes 4 and 5: Positive samples.

This study was performed on 3700 blood samples collected from patients attending the Rafsanjan blood transfusion services. All of the samples were found to be negative for HBsAg, anti-HCV, anti-HTLV-1 and anti-HIV antibodies. Out of 3700 samples, 352 (9.5%) were positive for anti-HBc out of whom 57 were found positive for HBV-DNA (Figure 1); These 57 HBsAg-/anti-HBc(+)/HBV-DNA(+) patients were diagnosed as OBI. Results of this study indicated that 16.1% of HBsAg-negative but anti-HBc-positive samples had detectable HBV-DNA which is 1.54% (57 of 3700) of the total collected samples. The mean±SD age of patients and controls were 28±6 and 28±8 years, respectively; there was no statistically significant difference in age between the two groups (Table 2). Three (3%) of the control group members were female and 97 (97%) were male while two of the patients (4%) were female and 55 (96%) were male. In addition, analysis of socio-economic conditions showed that there was also no significant difference between the patient and control groups (Table 2). Evaluation of the polymorphisms at 670 position of Fas showed that the frequency of ‘A' and ‘G' alleles were 69 (60.5%) and 45 (39.5%) in patients, respectively; the values were 124 (62%) and 76 (38%) in controls, respectively. Statistical analysis of these alleles indicated that the differences were not statistically significant (p = 0.810) (Table 3). Our results also showed that the prevalence of A/A genotype within the 670 region of Fas was 18 (32%) in patients and 40 (40%) in controls; the frequency of A/G genotype was 33 (58%) and 44 (44%) in patients and controls, respectively; and the frequency of G/G genotype in patients was 6 (11%) and in controls was 16 (16%) (Table 3). Statistical analysis of our data could not show any significant difference between the two groups regarding the frequencies of these genotypes (p = 0.232).

**Table 2:** Demographic and socioeconomic conditions of OBI patients and controls

**Variant**		**Healthy control **(n = 100)	**Patient** (n = 57)
**Age** (Mean ± SD)(Year)		28 ± 8	28 ± 6
**Sex**	**Female**	3 (3%)	2 (3.5%)
	**Male**	97 (97.8%)	55 (96.5%)
**Socio-economic status**	**Weak**	22 (22%)	12 (21%)
	**Medium**	47 (47%)	28 (49%)
	**High**	31 (31%)	17 (30%)

**Table 3:** The frequency of the 670 A→G polymorphisms within the Fas gene promoter of OBI patients and controls

		**PatientsNo.(%)**	**ControlNo.(%)**	**p-value**
**Genotype**	**A/A n **	38 (67%)	40 (40%)	p = 0.232
	**A/G n **	33 (58%)	44 (44%)	
	**G/G n **	6 (11%)	16 (16%)	
**Alleles**	**A n **	69 (60.5%)	124 (62%)	p = 0.810
	**G n **	45 (39.5%)	76 (38%)	

## Discussion

It is now well established that following viral hepatitis, some of the infected hepatocytes express the Fas and FasL system [15][20]. It is also stated that the rate of expression of FasL is correlated with the kind of clinical presentation and also correlates with the different stages of the HBV infection and associated liver disease [21]. Furthermore, Fas/FasL system may play a key role in apoptosis of the infected hepatocytes [22]. For example, over-chronic HBV and HCV infection [15] and the fulminant form of HBV infection [24] were reported. It seems that over-expression of Fas/FasL led to hepatocyte injury in hepatitis [23][24]. However, despite evidence suggesting a potential correlation between Fas/FasL system and disease status, our results showed that the frequency of evaluated alleles and genotypes was not different between OBI patients and healthy controls. Therefore, it can be concluded that these polymorphisms are not associated with OBI. Previous studies showed that the level of expression of Fas and FasL was associated with the clinical pattern of the disease [15][25]. For example, Bortolami et al. reported more expression of Fas and FasL in HBV-infected hepatocytes from patients with cirrhosis than in patients with chronic hepatitis [15]. Studies also reported that polymorphisms in the promoter regions of Fas and FasL influence the pattern of their expression [26]. Since OBI patients are unable to clear HBV completely, it could be suggested that the expression of Fas and FasL in OBI patients may be compromised by mechanisms other than polymorphisms within the promoter region. Despite evidence linking a functional role for Fas to progression of viral hepatic disease, our data showed that the polymorphisms, which are known to influence Fas expression levels, were not statistically different between OBI patients and healthy controls. Further studies should be done on the expression of Fas at the protein level to confirm this. To our knowledge, this is the first report which investigates the involvement of Fas polymorphisms with OBI. However, in related research, Jung et al. also showed that there were no significant associations between FasL (844 C/T) polymorphism and HBV clearance in chronic hepatitis patients [22]. Interestingly, similarly to our own findings, Sung and colleagues could not find a significant difference between the healthy donors and patients with hepatocellular carcinoma [21]. Similarly, several studies reported a correlation between Fas and FasL polymorphisms during HCV infection [27][28][29]. One reason for the discrepancy between our results and these studies could be explained by the different types of hepatitis infection (HCV) and there may also be genetic differences in the populations studied. In addition, there may also be subtle differences in the type of disease from our studied population. It is not clear through what mechanisms OBI patients are unable to completely overcome the viral contamination, however, based on the current studies it seems that the polymorphisms within the promoter region of the Fas were not correlated with OBI. Finally, due to the complexity of OBI, other aspects of the disease are needed to be examined. For instance our previous study showed that the serum level of IL-17, an inflammatory cytokine, was increased, whereas the serum level of IL-10 has decreased in OBI patients [11]. Therefore, our future studies will explore polymorphisms and the expression levels of these and other important cytokines and their receptors within the OBI patients.
